# Extreme natural size variation in both sexes of a sexually cannibalistic mantidfly

**DOI:** 10.1098/rsos.220544

**Published:** 2022-08-17

**Authors:** Laurel B. Lietzenmayer, Lauren M. Goldstein, Josephine M. Pasche, Lisa A. Taylor

**Affiliations:** ^1^ Entomology and Nematology Department, University of Florida, Gainesville, FL 32611, USA; ^2^ Department of Entomology, Michigan State University, East Lansing, MI 48824, USA

**Keywords:** egg predator, spider, sexual cannibalism, body size, museum collections

## Abstract

In sexually cannibalistic animals, the relative sizes of potential mates often predict the outcome of aggressive encounters. Mantidflies are spider egg predators as larvae and generalist predators as adults. Unlike most cannibalistic species, there is considerable individual variation in body size in both sexes. Using preserved collections of *Dicromantispa sayi*, we focused on three body size metrics that we found to be positively correlated and accurately measured across researchers. We found extreme size variation in both sexes: the largest 10% of females were 1.72× larger than the smallest 10%, and the largest 10% of males were 1.65× larger than the smallest 10%. On average, females were 7.94% larger than males. In exploring possible causes of this variation, we uncovered differences among populations. To explore the effect of spider egg sac size on adult mantidfly size, we reared mantidfly larvae on egg sacs from two jumping spider species with small or large egg sacs. Mantidfly larvae reared on small egg sacs were smaller than those reared on large egg sacs. This study provides the groundwork to design ecologically relevant experiments exploring the causes and consequences of extreme size variation in an understudied system with intriguing natural history.

## 1. Introduction

Body size plays a key role in an organism's life history and can be shaped by many factors [1]. Adult body size is tightly connected to physiological constraints and natural selection (e.g. predation, foraging success), but sexual selection can shape female and male body size in different ways depending on the specific selection pressures and life history strategies present in a system [2–4]. The relationship between the size differences of females and males in a natural population termed sexual size dimorphism (SSD), typically follows patterns of female-bias (females larger than males) or male-bias (males larger than females) [3]. Several non-mutually exclusive hypotheses exist to explain why certain taxa exhibit one type of SSD or the other, focusing on factors that maximize reproductive output. For example, females are usually larger in systems where females invest more in egg production (e.g. reptiles: [5,6]), or where smaller males are more efficient at finding mates (e.g. spiders: [7]; insects: [8]). By contrast, males are usually larger in species where male size determines the outcome of male–male contests (e.g. mammals: [9]; insects: [10–12]).

Sexual size dimorphism becomes particularly interesting in systems where aggression and cannibalism are intertwined with courtship. In many cannibalistic species, being smaller than your mate increases your risk of being cannibalized [13]. Research investigating the relationship between SSD and cannibalistic behaviours has mainly focused on spiders and praying mantises, mostly with strong female-biased SSD and cannibalistic females (spiders: [13–16]; mantids: [17,18]). Less work has been done on species with male-biased SSD and the occurrence of males cannibalizing females [19,20]. However, in well-studied cannibalistic systems, the overall degree and direction of SSD are relatively stable and allow for scientists to describe specific species as exhibiting largely female-biased or male-biased SSD. Cannibalistic species that exhibit extreme size variability in both sexes are rare and less well studied. In these species, either the male or female in any mating pair could be significantly larger than the other, meaning that either sex can be at risk of cannibalism. Studying how size affects reproductive behaviour, aggression and cannibalism in systems with extreme size variation in both sexes may provide new insight into the evolution and function of SSD across taxa.

**Figure 1.**
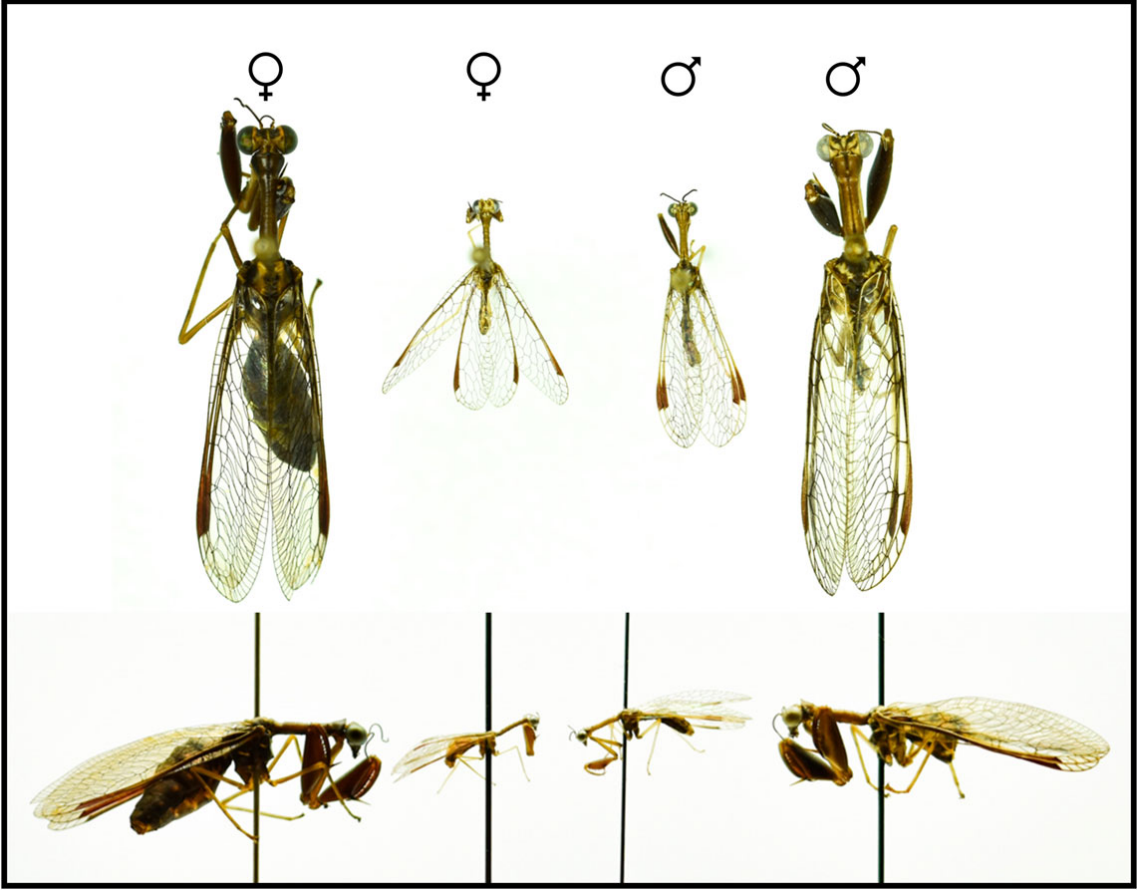
Field-collected adult female and male *Dicromantispa sayi* specimens depicting the extreme size variation in both sexes. The top row of images shows the dorsal view, while the bottom row of images shows the lateral view of the same specimens.

One of these fascinating and overlooked groups is the mantidflies (Neuroptera: Mantispidae). Adult mantidflies (Neuroptera: Mantispidae) are highly visual and voracious predators with elaborate courtship displays and both sexes can be aggressive or cannibalistic during courtship [21–23]. Regardless of sex, adult mantidflies can vary considerably in size (figure 1) and are therefore an ideal system to use in studying the role of variable SSD on courtship and sexual cannibalism [22,24,25]. The intriguing natural history of this system may play a key role in the size variation we observe in adults. Mantidfly larvae specialize on spider eggs from a wide range of spider taxa [25,26]. They find spider eggs either by climbing aboard spiders and entering egg sacs as they are constructed or by seeking out egg sacs and burrowing into them directly [22]. While boarded on spiders, mantidfly larvae can maintenance feed on haemolymph from their spider host [27]. Mantidfly larvae that successfully locate and burrow into egg sacs directly may be able to slow or halt the development of the spider eggs within the egg sac via an aggressive allomone, allowing them to feed on more eggs before they develop into spiderlings [28]. An adult mantidfly's size is thought to be dependent on these resources available during development (i.e. the eggs within the spider egg sac that a larva feeds on), as larvae are completely restricted to the resources within the egg sac they enter [25]. In nature, spider egg sacs vary from a few eggs to several hundred eggs [29,30]. This variation may be what drives the remarkable size variation of adult mantidflies, as first noted and tested by Redborg & Macleod [25] using manipulated egg sacs and varying amounts of spider eggs. Although currently unknown, other selection pressures, particularly sexual selection, may also contribute to body size variation in mantidflies.

*Dicromantispa sayi* (Banks, 1897) (and other mantidfly species) are geographically distributed across a wide range of habitats with varying seasonalities, temperatures, elevations and spider communities [31–33]. These variable ecological factors could be potential drivers of the differences in body sizes between mantidflies. Previous studies that have directly measured body size in large samples of mantidflies are limited, and only include individuals from a relatively small geographical range in the midwestern United States ([25]; but see data included in Bakkes *et al*. [24] for a small sample of males of two African species). Little is known about how body size varies across wide geographical ranges and among geographically distinct populations. Such knowledge would be a valuable prerequisite for systematically testing broader ecological theory about what drives animal size and shape (e.g. Bergmann's rule, Allen's rule, etc.).

Currently, to our knowledge, there are no empirical studies that quantify the extent of body size variation and SSD in natural mantidfly populations across a wide geographical range. This is a necessary first step for designing ecologically relevant and rigorous manipulative experiments to better understand the relationship between mating behaviour and SSD in mantidflies. Using the mantidfly, *D. sayi*, our study had the following four objectives: (1) First, we measured three body size metrics in preserved field-collected individuals to determine whether they could be measured easily and repeatably across researchers, as well as determine whether these size metrics were correlated with one another in a way that would allow them to be used interchangeably in studies of natural body size variation in this species. (2) Second, we quantified and compared natural body size variation in males and females. We then explored two hypotheses that may explain the source of variation observed. (3) To explore whether factors related to geography might contribute to this extreme variation, we compared body size data among three distinct collection sites in the southeast USA for which we had the largest numbers of specimens available. (4) Finally, to explore whether the size of the particular spider egg in which a mantidfly larva develops might contribute to adult body size variation, we reared live mantidfly larvae on eggs from two different jumping spider species that produce egg sacs of markedly different sizes. We then assessed whether mantidflies reared on larger egg sacs matured into larger adults (compared with those reared on smaller egg sacs).

## 2. Methods

### 2.1. Objective 1: assessing repeatability and correlations among body size metrics

**Figure 2.**
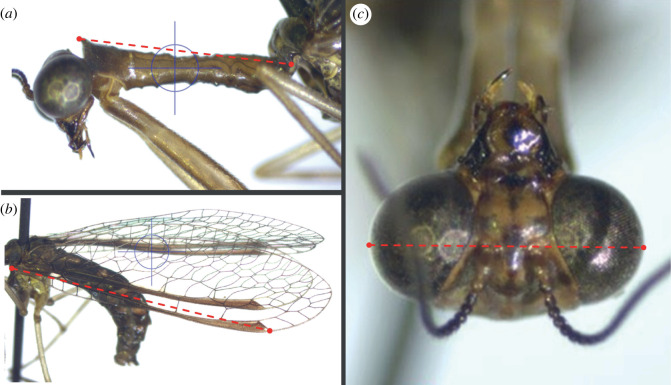
Three body size measurements measured from images of *Dicromantispa sayi* museum specimens, indicated by the dashed line. These measurements are referred to as (*a*) pronotum length, (*b*) subcostal vein length and (*c*) head capsule width.

We targeted three body parts originally characterized by Redborg & Macleod [25] and adapted them to ensure precision during imaging and measuring: head capsule width (measured through eyes at base of antennae from anterior viewpoint), pronotum length (lateral view from posterior point of articulation to articulation point with mesothorax), and subcostal vein length (base of subcosta to most distal corner of darkened pterostigma) (figure 2 for examples of images and measurements).

To assess whether these metrics could be measured repeatably across multiple researchers, we randomly selected ten *D. sayi* specimens from the Florida State Collection of Arthropods at the Division of Plant Industry (Gainesville, FL, USA) and three researchers measured each specimen independently for each of the three body size metrics while viewing them through a microscope camera (Zeiss, Axiocam 105 colour, White Plains, NY) and Zen 2 lite software (Zeiss, White Plains, NY).

To assess correlations across these three body size metrics, we measured all *D. sayi* specimens available in the Florida State Collection of Arthropods (*n* = 255). Because each body size metric was highly repeatable across researchers in the first part of this objective described above (see Results), only one researcher measured each specimen for this part of the study. Measurements were omitted when one or more measurement points were not visible on a specimen due to damaged structures or another body part obscuring its view.

### 2.2. Objective 2: quantifying body size variation and sexual size dimorphism

To examine sex-specific patterns of body size variation, we determined the sex of each individual specimen measured in Objective 1 (*n* = 255) by inspecting the ventral terminalia [34].

### 2.3. Objective 3: assessing geographical differences in body size

To investigate whether size distributions were different for mantidflies collected from distinct geographical sites, we recorded all collection locality information from labels for the specimens described above (*n* = 225). Geographical sites included three parks and biological stations across the southeast USA: Archbold Biological Station (Venus, FL, USA), Lake Corpus Christi State Park (Mathis, TX, USA), and Stephen Foster State Park (Fargo, GA, USA). Sites were selected for comparison if they included at least 25 individual mantidflies.

### 2.4. Objective 4: establishing the effect of spider egg sac size on adult mantidfly body size

To explore the idea that variation in spider egg sac size might drive variation in adult mantidfly body size, we reared individual mantidfly larvae on egg sacs of different sizes. We used egg sacs from two locally abundant jumping spider species whose ranges overlap with *D. sayi*: *Habronattus trimaculatus* and *Phidippus regius*. *Habronattus* egg sacs are considerably smaller on average compared to *Phidippus* egg sacs (approx. 2–3% of mass; [29]; LAT unpublished data), which suggests *Habronattus* egg sacs likely have less nutrient content available compared to *Phidippus* egg sacs [29,30,35–37].

To generate spider egg sacs for rearing mantidfly larvae, we collected adult female *H. trimaculatus* (*n* = 34) and *P. regius* (*n* = 21) via sweep net and hand collection from various locations in north-central Florida (USA) from May–September 2019. We housed spiders in individual acrylic boxes (10.16 × 10.16 × 12.86 cm) with a mesh hole for ventilation. We fed each species a diet of appropriately sized crickets (*Gryllodes sigillatus*; pinheads for *H. trimaculatus*, larger juveniles for *P. regius*) approximately equal to their body weight three times per week and sprayed each box with water daily. We monitored spiders for newly laid egg sacs and only used the first clutch of eggs from each female.

We hand-collected field-mated adult *D. sayi* females (*n* = 7) from lights at various locations in north-central Florida (USA) from May–September 2019 and housed them in the laboratory in individual acrylic boxes (10.16 × 10.16 × 12.86 cm) with a mesh hole for ventilation. We fed adult female mantidflies approximately 10 fruit flies (*Drosophila melanogaster*) and provided a spray of water daily. After laying a clutch of eggs on the wall of the box, females were rehoused to allow for the laying of additional clutches. We sprayed the enclosures of egg clutches with water and monitored them daily for hatching.

Individual newly hatched first instar mantidfly larvae were randomly assigned to a spider species (*H. trimaculatus* or *P. regius*) and were placed directly onto the outer silk case of a 1–3 day old egg sac (one larva per egg sac). This allowed them to penetrate the egg sac to enter it and feed on the eggs to continue development. We monitored egg sacs daily for adult emergence. Adult mantidflies raised on these egg sacs (*H. trimaculatus*: *n* = 9; *P. regius*: *n* = 6) were housed as described above and allowed to naturally expire in the laboratory. We then pinned the mantidfly specimens and measured body size metrics (i.e. head capsule width, pronotum length, subcostal vein length) as described above.

### 2.5. Statistical analyses

All data were analysed in RStudio using R v. 4.0.3 [38]. Unless otherwise noted, all relevant statistical assumptions were met; in cases where assumptions were not met, we used data transformations or non-parametric alternatives, as described below. Raw data are archived on Dryad (https://doi.org/10.5061/dryad.mkkwh712n).

#### 2.5.1. Objective 1

To assess whether the measurements for each body size metric (i.e. head capsule width, pronotum length, subcostal vein length) were repeatable across researchers, we used linear regressions to determine if the measurements correlated across three different researchers. For each metric, we examined relationships for each possible pairwise combination of researchers (researcher 1 versus researcher 2, researcher 1 versus researcher 3, researcher 2 versus researcher 3). We then used the averaged *R*^2^ values of each regression to rank the repeatability of each metric.

To evaluate whether any of the three metrics could be used interchangeably as a proxy for body size, we conducted linear regressions on data from all specimens (*n* = 255) to determine how tightly the three size metrics positively correlated with each other.

Measurements were only included in the above analyses if all three body size metrics were successfully measured for the specimen. All linear regression models were performed with the lm() function in *R*.

#### 2.5.2. Objective 2

For each of the three body size metrics, we used two sample *t*-tests to determine if males and females differed in size. We calculated the sexual size dimorphism index (SDI) for each body size metric using the equation SDI = (average size of the larger sex/average size of the smaller sex) − 1. We assigned positive values to indicate females as the larger sex and negative values to indicate males as the larger sex. This index, recommended by Lovich & Gibbons [39], centralizes around 0 (i.e. sexes identical in size).

We used Levene's tests to determine if the sexes differed in how much size variation was present in the three body size metrics (using the leveneTest() function in the car package; [40]). We calculated the coefficient of variation (CV) for each body size metric for each sex by dividing the standard deviation by the mean, then multiplying by 100.

To aid in describing how extreme the size variation may be among individuals in a population, and therefore enable us to estimate how often different-sized individuals might encounter each other in courtship interactions in nature, we calculated summary statistics (i.e. mean ± standard deviation, range). We also calculated the percent increase in size between the smallest 10% to the largest 10% of the distribution for females and males. To broaden the pool of individuals considered in these comparisons, we also included the percent increase from the smallest 25% and the largest 25% of the distribution for females and males.

#### 2.5.3. Objective 3

We used three separate linear models using the lm() function in *R* (one for each body size metric) to compare size differences between populations and sexes. The size measurement was the response variable and the explanatory variables were population (i.e. Archbold, Corpus Christi, Stephen Foster) and sex. We included an interaction term for population and sex to determine if females and males were different in size within each population. If significance was detected from the Anova() function in the car package [40], we performed a Tukey multiple comparison test with adjustment for multiple testing using the emmeans() function in the emmeans package [41].

We used Levene's tests to determine if the populations differed in how much size variation was present (using the leveneTest() function in the car package; [40]). The data for the model with subcostal vein length as the response variable were log-transformed because they did not meet linear model assumptions. We calculated the coefficient of variation (CV) for each body size metric, as described above.

#### 2.5.4. Objective 4

To determine if mantidflies raised on *H. trimaculatus* egg sacs were smaller than those raised on *P. regius* egg sacs, we used non-parametric Mann-Whitney *U* tests with our three size measurements as response variables and spider egg species (i.e. *H. trimaculatus* or *P. regius*) as explanatory variables. We used non-parametric tests because the size distributions for all body size estimates did not meet the assumptions of a two-sample *t*-test.

## 3. Results

### 3.1. Objective 1

All three body size metrics were highly repeatable across researchers, with head capsule width (*n* = 10) and pronotum length (*n* = 10) showing the strongest positive correlations among researchers (*R*^2^ = 0.98, 0.97, respectively) compared to subcostal vein length (*n* = 10; *R*^2^ = 0.87).

All three body size metrics were good, interchangeable proxies for overall body size of mantidflies that strongly positively correlated with each other (table 1; electronic supplementary material, figure S1).

**Table 1.** Linear regression results for all possible combinations of the three body size measurements for *Dicromantispa sayi* specimens. Note that sample sizes shown here are less than 255 (the total number of specimens available) because they exclude those specimens for which one or both measurement points were not visible. Significant *p*-values are shown in bold.

	*R*^2^	*p*	coefficient estimate
pronotum length versus head capsule width (*n* = 202)	0.86	<0.000001*	0.56
subcostal vein length versus head capsule width (*n* = 202)	0.90	<0.000001*	0.21
subcostal vein length versus pronotum length (*n* = 202)	0.85	<0.000001*	0.34

### 3.2. Objective 2

**Figure 3.**
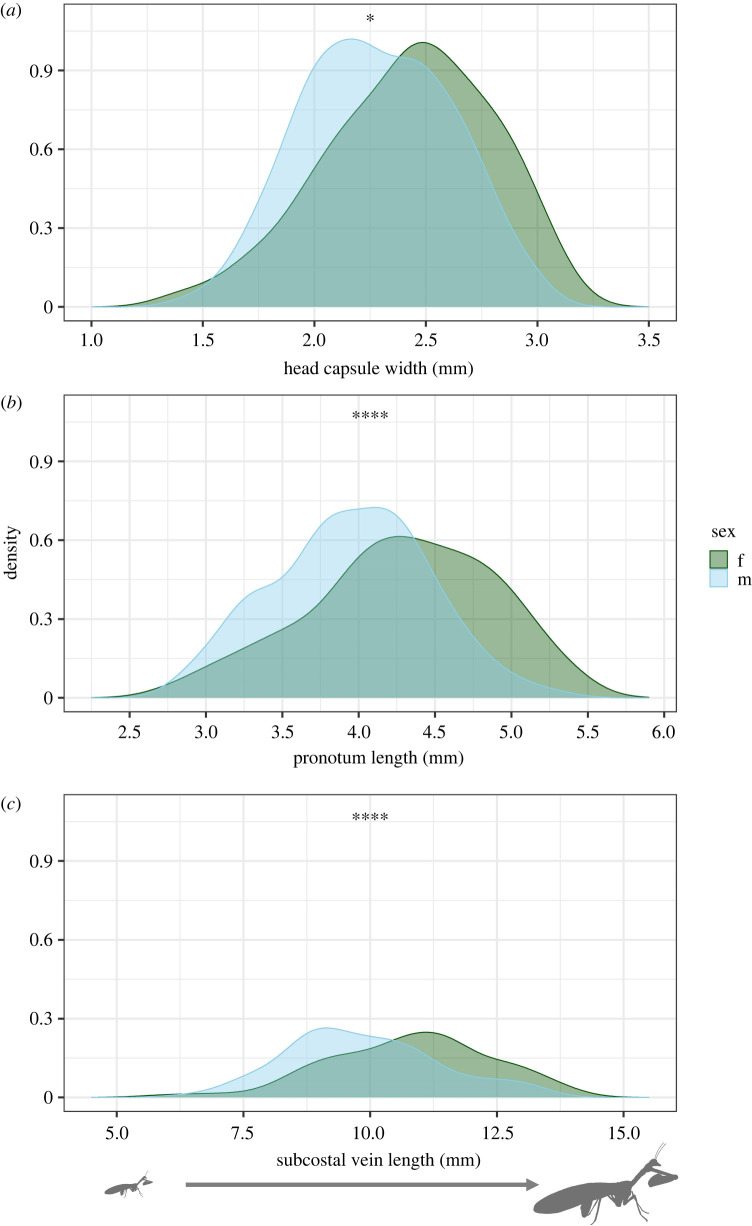
Comparisons of size distributions between *Dicromantispa sayi* females and males for three different body size measurements; (*a*) head capsule width, (*b*) pronotum length and (*c*) subcostal vein length. Asterisks indicate significant sex differences in size (*p < 0.01; ****p < 0.0001).

Females, on average, were approximately 7.94% larger than males in head capsule width (*t*_242_ = 3.21, *p* = 0.0015), pronotum length (*t*_226_ = 4.57, *p* < 0.00001), and subcostal vein length (*t*_236_ = 4.62, *p* < 0.00001) (table 2 and figure 3). Means, 95% confidence intervals (CI), and sexual dimorphism index (SDI) values are listed in table 2.

**Table 2.** Comparisons between female and male *Dicromantispa sayi* specimens for three body size metrics (head capsule width, pronotum length, and subcostal vein length). Significant *p*-values are shown in bold.

	mean ± s.e.	95% CI	*t*	*p*	sexual dimorphism index (SDI) [(female size/male size) − 1]
head capsule width			3.21	0.0015*	0.061
female (*n* = 123)	2.42 ± 0.03	[2.36–2.49]			
male (*n* = 121)	2.28 ± 0.03	[2.22–2.34]			
pronotum length			4.57	<0.00001*	0.084
female (*n* = 114)	4.28 ± 0.06	[4.18–4.38]			
male (*n* = 114)	3.95 ± 0.05	[3.84–4.05]			
subcostal vein length			4.62	<0.00001*	0.093
female (*n* = 121)	10.8 ± 0.15	[10.5–11.1]			
male (*n* = 117)	9.86 ± 0.14	[9.57–10.1]			

Variation in body size was similar for both females and males for head capsule width (coefficient of variation (CV): female = 15.4; male = 14.5; *F*_1,242_ = 0.98, *p* = 0.323), pronotum length (CV: female = 14.0; male = 12.8; *F*_1,226_ = 3.16, *p* = 0.077), and subcostal vein length (CV: female = 14.8; male = 15.1; *F*_1,236_ = 0.39, *p* = 0.533) (figure 3). Size comparisons between the smallest 10% and largest 10% (and additionally the smallest 25% and largest 25%) of specimens measured are listed in table 3.

**Table 3.** Means ± standard deviation and ranges for the largest and smallest 10% and 25% of *Dicromantispa sayi* specimens (for subset females and subset males). The far-right columns give a percent increase in size for the smallest 10% and 25% of males to the largest 10% and 25% of females and vice versa. Percent increase = [(largest X% - smallest X%) / smallest X%] × 100. For example, the largest 10% of female head capsule widths are 73.16% larger than the smallest 10% of male head capsule widths.

	mean ± s.d. (mm)					% increase	
	largest 10%	smallest 10%	largest 25%	smallest 25%	range (mm)	10%	25%
						largest ♀, smallest ♂	largest ♀, smallest ♂
♀ head capsule width (*n* = 123)	2.98 ± 0.08	1.73 ± 0.17	2.87 ± 0.11	1.93 ± 0.21	1.74	73.16	54.48
♀ pronotum length (*n* = 114)	5.19 ± 0.14	3.17 ± 0.21	5.00 ± 0.19	3.49 ± 0.32	2.62	67.44	52.10
♀ subcostal vein length (*n* = 121)	13.33 ± 0.35	7.87 ± 0.94	12.75 ± 0.59	8.68 ± 0.92	8.01	78.50	57.44
						largest ♂, smallest ♀	largest ♂, smallest ♀
♂ head capsule width (*n* = 121)	2.83 ± 0.09	1.72 ± 0.11	2.71 ± 0.12	1.86 ± 0.14	1.51	63.42	40.46
♂ pronotum length (*n* = 114)	4.79 ± 0.21	3.10 ± 0.14	4.57 ± 0.24	3.29 ± 0.20	2.37	51.38	31.01
♂ subcostal vein length (*n* = 117)	12.71 ± 0.43	7.47 ± 0.48	11.85 ± 0.83	8.10 ± 0.64	7.02	61.59	36.49

### 3.3. Objective 3

**Figure 4.**
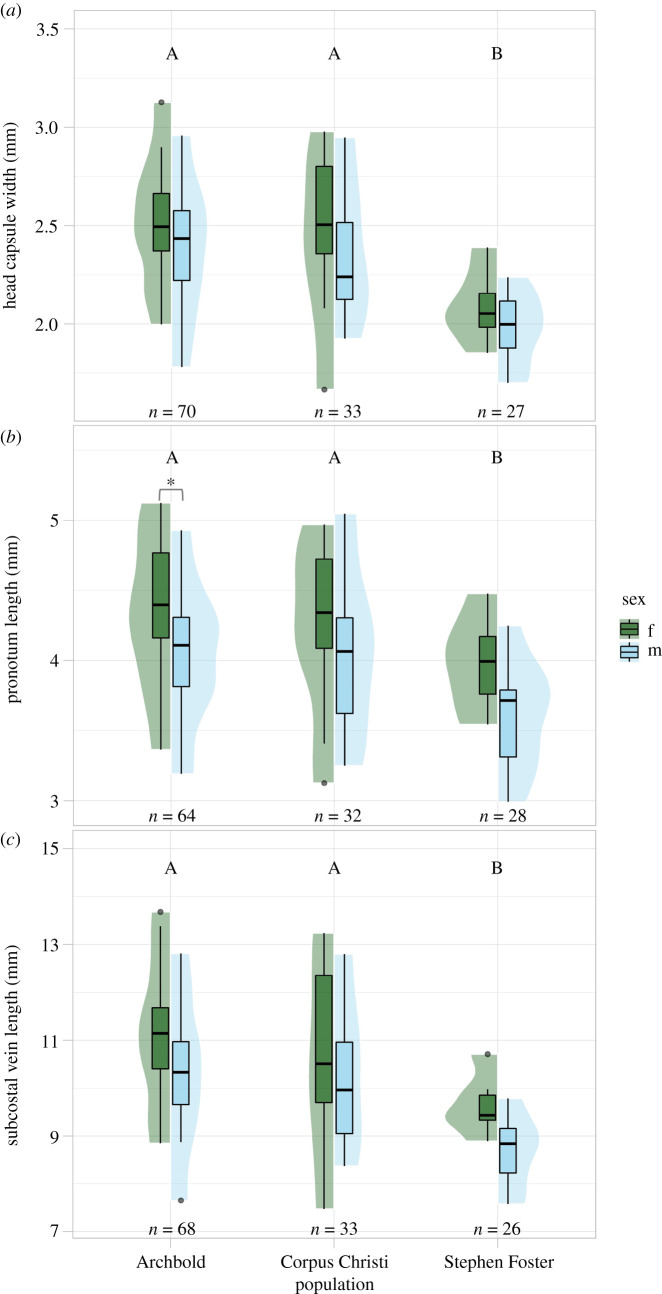
Comparisons of size distributions between *Dicromantispa sayi* populations (Archbold Biological Station, Corpus Christi State Park, Stephen Foster State Park) for three different body size measurements; (*a*) head capsule width, (*b*) pronotum length and (*c*) subcostal vein length. Females are indicated by dark green boxes and males are indicated by light blue boxes. Shaded areas around box plots represent the density curves of each distribution. Different letters (A,B) above each pair of boxplots specify differences in size among populations, *p* < 0.05. Brackets and asterisks (*) indicate differences between sexes within populations, *p* < 0.05.

Linear models comparing mantidfly body size across populations and sexes showed that populations differed in size (table 4). Across all body size metrics, mantidflies from Archbold Biological Station and Corpus Christi State Park were similar in size (table 4 and figure 4). Mantidflies from Stephen Foster State Park were smaller on average than mantidflies from Archbold (table 4) and Corpus Christi (table 4 and figure 4). Overall, females were larger than males for all body size metrics (table 4). However, pairwise comparisons between females and males within each population show this statistical difference in size is lost for all size metrics and all populations except for the pronotum length of mantidflies from Archbold Biological Station (*t*_118_ = 3.196, *p* = 0.022; figure 4*b*)**.** There was no significant interaction between sex and population for any body size metric (table 4).

**Table 4.** Full model and pairwise comparison statistics for analyses in Objective 3 comparing body size of *Dicromantispa sayi* adult females and males from three distinct populations in southeast USA. Significant *p*-values are shown in bold.

	*F*	*t*	df	*p*
population				
head capsule width	24.33		2, 124	**<0.0001**
Archbold-Corpus Christi		0.582		0.801
Archbold-Stephen Foster		6.641		**<0.0001**
Corpus Christi-Stephen Foster		5.354		**<0.0001**
pronotum length	10.01		2, 118	**<0.0001**
Archbold-Corpus Christi		0.624		0.808
Archbold-Stephen Foster		4.087		**0.0002**
Corpus Christi-Stephen Foster		3.143		**0.006**
subcostal vein length	17.03		2, 121	**<0.0001**
Archbold-Corpus Christi		1.279		0.410
Archbold-Stephen Foster		5.401		**<0.0001**
Corpus Christi-Stephen Foster		3.771		**0.001**
sex				
head capsule width	6.80		1, 124	**0.010**
pronotum length	17.91		1, 118	**<0.0001**
subcostal vein length	9.26		1, 121	**0.003**
population X sex				
head capsule width	0.136		2, 124	0.873
pronotum length	0.061		2, 118	0.941
subcostal vein length	0.182		2, 121	0.834

Size variation was similar for all populations of mantidflies for pronotum length (coefficient of variation (CV): archbold = 11.1; Corpus Christi = 12.7; Stephen Foster = 9.8; *F*_2,121_ = 1.58, *p* = 0.210) and subcostal vein length (CV: archbold = 11.3; Corpus Christi = 14.4; Stephen Foster = 8.5; *F*_2,125_ = 0.13, *p* = 0.721). However, when considering head capsule width, we found that mantidflies from Stephen Foster State Park were less variable in size than those from Archbold or Corpus Christi State Park (CV: archbold = 10.9; Corpus Christi = 13.6; Stephen Foster = 8.4; *F*_2,127_ = 5.808, *p* = 0.004).

### 3.4. Objective 4

**Figure 5.**
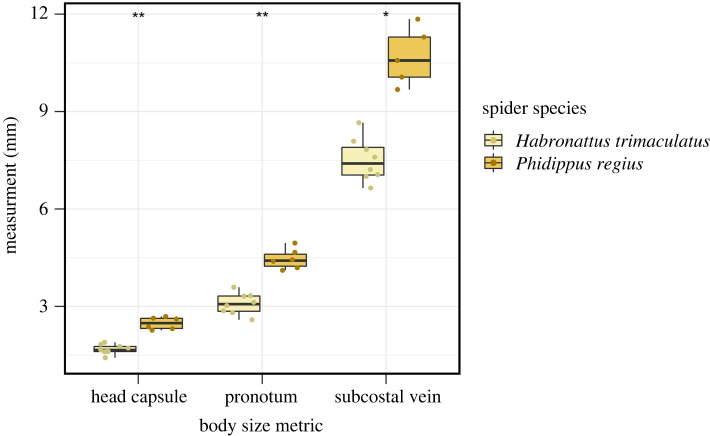
Adult mantidfly, *Dicromantispa sayi*, body sizes from individuals raised on egg sacs of the relatively small *H. trimaculatus* versus egg sacs of the relatively large *P. regius*. Body size metrics include head capsule width, pronotum length, and subcostal vein length. ‘*’ indicates *p* < 0.01; ‘**’ indicates *p* < 0.001.

As expected, mantidfly larvae raised on *H. trimaculatus* egg sacs were smaller than those raised on *P. regius* egg sacs in head capsule width (*w* = 0, *p* = 0.0004), pronotum length (*w* = 0, *p* = 0.0007) and subcostal vein length (*w* = 0, *p* = 0.0016; figure 5).

## 4. Discussion

The unique natural history of mantidflies and their extreme variation in body size shown here provides a new and interesting perspective into how natural and sexual selection can interact to influence morphology. After first establishing that three body size metrics (head capsule width, pronotum length, subcostal vein length) can be measured easily, repeatably across researchers and strongly positively correlate with each other, we went on to use these metrics to quantify body size variation using specimens from across southeast USA. We found that both sexes were extremely variable in size, with females being larger on average than males. When comparing mantidflies from three distinct geographical sites, we found intriguing differences in body size and the degree of size variation. This geographical variation in the body size of mantidflies can be used to guide our hypotheses about the causes of body size variation. Finally, we provide evidence that mantidfly adult body size is affected by the size of the spider egg sac in which the mantidfly completes development. Taken together, these data provide a rich context for further exploration of the causes and consequences of extreme body size variation in mantidflies. Moreover, they provide the concrete data needed to design ecologically relevant manipulative experiments.

### 4.1. Causes of extreme variation in mantidfly body size

One explanation for the cause of extreme body size variation is the larval mantidfly's spider egg predator strategy (also speculated by Redborg & Macleod [25]). Because spider egg sacs vary widely in size and energy content [29], the egg sac in which a mantidfly larva finds itself may be the key determinant of its future adult body size. This idea has been supported by work by Redborg & Macleod [25], who provided mantidfly larvae with different numbers of eggs from the common house spider (*Achaearanea tepidariorum)* and found that individuals provided with more eggs were larger as adults*.* Redborg's experiments used ‘pseudo egg sacs’ (eggs that were removed from egg sacs and provided to mantidfly larvae in drilled holes within a plaster substrate and covered with a glass coverslip). Our results using natural egg sacs from two species of jumping spiders support this idea as well; adult mantidflies raised on the small egg sacs of *Habronattus trimaculatus* were considerably smaller than those raised on the larger eggs sacs of *Phidippus regius*. While the effects we observed seem likely to be driven by egg sac size, we cannot rule out other factors that differ between the two spider species, such as species-specific differences in egg or egg sac structure, egg size and/or nutritional content that may affect how well these eggs support a mantidfly larva's growth. Even though our data do not allow us to isolate the effect of egg sac size from other factors, it is clear that the species producing the egg sac in which a mantidfly develops has an important impact on their adult body size in some capacity. Previously established techniques for rearing mantidflies using pseudo spider egg sacs can be used to tease apart some of these and other factors because they allow us to directly manipulate variables like spider egg number, egg size, egg species or even the developmental state of eggs [25,42]. Now that we have extensive data on natural variation in mantidfly body size, we can assess whether such egg manipulations result in patterns of size variation that mirror what we see in natural populations.

The differences we observed between the three distinct mantidfly geographical sites in our study in both overall body size and the extent of variation in body size can also help us generate additional hypotheses. Some of the limitations to using specimens from collections may also simultaneously aid in informing what direction of inquiry to take first. The mantidfly specimens used in this study are limited to those collected and donated over several decades, resulting in biases in the timing of collection listed on specimen labels. For example, all specimens from Stephen Foster were collected by the same person in the month of September of the same year, but specimens from Archbold and Corpus Christi were collected by various people over a wider span of seasons and years. While this makes meaningful comparisons across the populations more difficult, it can also allow us to speculate as to why size distributions are different. The smaller and less variable body sizes of mantidflies from Stephen Foster may be the result of multiple non-mutually exclusive factors, including differences in local spider communities and the spider eggs available to mantidfly larvae [33,43], seasonality/temperature/precipitation (e.g. abiotic factors that also affect body size in other insects: [44–46]), and microhabitats that larvae must traverse to find spiders [47,48]); all of these ideas can now be explored further. As a starting point, we examined but did not find any noticeable anomalies in historical climate data for Stephen Foster in September of 1978 (or the three months preceding this when these mantidflies would have been undergoing development) compared to other years (five preceding and five following 1978; accessed through NOAA climate data online database: https://www.ncdc.noaa.gov/cdo-web/). More work is clearly needed to better understand how and why body size varies with geography, if indeed these patterns hold up with more data. In another mantidfly species from Africa, extreme body size variation was reported that appeared to be independent of geography, but this study was limited to a relatively small sample size of only male specimens [24].

### 4.2. Consequences of extreme variation in mantidfly body size

The preliminary work of assessing different metrics for measuring traits and determining their repeatability and ease of collection is an important first step that lays the foundation for future carefully designed experiments to examine the consequences of body size [48–51]. With robust body size metrics in hand, we can now more carefully and confidently investigate the role of body size in behavioural studies [52–54] and quantify sexual size dimorphism [3,4,16]. Moreover, the ability to use interchangeable body size metrics opens up the possibility of incorporating and combining data from images in digitized museum collections (e.g. [55], where measurements of specimens from all possible angles may not be possible). Well-established methods for measuring body size can also be used to assign individuals to certain size classes, provide confidence in treatments meant to alter body size in certain directions, and assess body size variation in specific natural populations to test ecological patterns on a broader scale than presented here. Additionally, in future studies using *D. sayi,* we will be able to estimate how likely it is that pairs of males and females of different sizes will encounter one another in the field and how often a male might encounter extremely large or extremely small females.

Data presented in this study (specifically those found in table 3) can be used in future research to manipulate mating pairs of various body sizes that reflect natural variation to ask specific questions about how body size affects aggression, cannibalism and mate choice. Because of their visual acuity [21], it may be possible for both mantispid males and females to visually assess a potential mate's size during courtship which may alter their behaviour depending on the relative size of the other individual. From the female's perspective, when a courting male is relatively small, it may be more beneficial to cannibalize and wait for a more ideal, larger male. Female mantidflies may prefer to mate with larger males because males produce nutrient-rich spermatophores that are transferred to females during copulation to aid in egg provisioning [25], and the size and quality of spermatophores may positively correlate with male size (observed in other arthropod systems; [49,50]). Alternatively, larger males may be more successful simply because they are riskier for females to attack or are better equipped to coerce mating attempts (as seen in a sexually cannibalistic mantid; [51]). From the male's perspective, being able to visually assess the relative size of a female may allow a male to avoid cannibalism by determining if the risk is too great to court a relatively larger female. However, spermatophore production and courtship displays may be extremely costly (as observed in other systems; [52,53]); if this is the case in mantidflies, it might result in males that are choosier. Males may be expected to prefer to mate with larger females if they produce larger, more successful offspring (common in arthropods; [54,55]). Mating with a larger female may also be advantageous if larger females have higher fecundity (common in many insect taxa; [54,56]), but courting a relatively large female would need to be weighed against the increased risk of being cannibalized. For both sexes, the optimal size of a partner likely depends on one's own size; as such, both small and large individuals of both sexes might mate successfully under the right circumstances, and this may contribute to the maintenance of size variation in natural populations.

The extent of size variation that we found in both sexes is considerable, particularly when we consider what this means for the likelihood of different-sized individuals encountering one another during courtship interactions in the field. Notably, for the smallest 10% of males sampled, approximately 36% of all females are at least 50% larger in head capsule width. For the smallest 10% of females sampled, approximately 19% of all males are at least 50% larger in head capsule width (range values and percent size increases in table 3). While not particularly common across the animal kingdom, this extreme degree of body size variation has been observed in some other insect systems [57,58]. Additionally, the body size variation documented here may follow similar patterns seen in some other animals whose body size is similarly constrained by resources during development (e.g. parasitoid insects; [59,60]). However, the mantidfly system is exceptionally unique due to extreme consequences of size differences in a mating context. For other animals, being small compared to other conspecifics may have costs, but being eaten by their mate is most likely not one of them.

**Figure 6.**
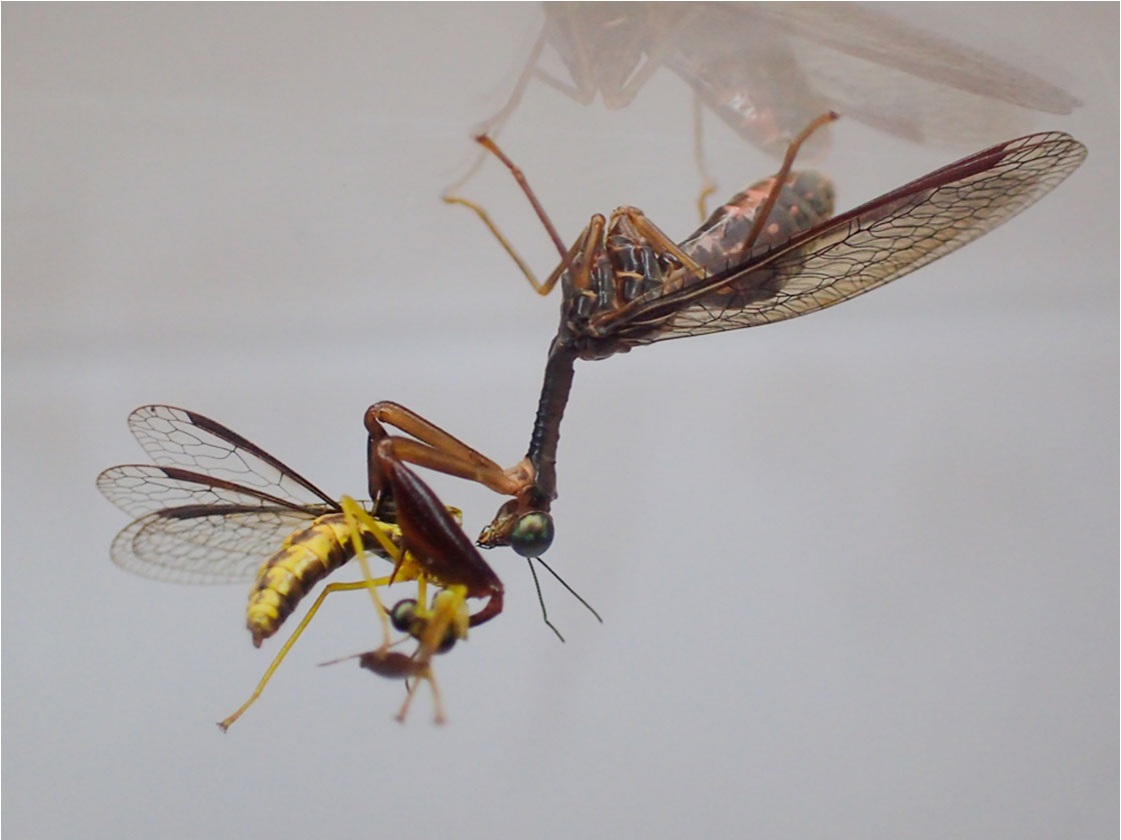
Adult female *Dicromantispa sayi* engaging in pre-copulatory sexual cannibalism of a male.

While examining mating interactions was not the focus of the present study, we also made several anecdotal behavioural observations between females and males of different body sizes to better inform future formal studies manipulating size in mating pairs. In these observations, one male and one female were placed in an arena overnight and video recorded under red light. In one instance, when the male was larger than the female, the female displayed an intriguing behaviour toward the male in which she faced the male, raised both pairs of wings perpendicular to the ground, and quickly approached the male (electronic supplementary material, video S1). This behaviour often ended with the female striking at the male with her raptorial forelegs and the male responding by striking back (electronic supplementary material, video S1). In the second instance, when the female was larger than the male, the male spent a considerable amount of time courting the female in a comparable manner described in Redborg & MacLeod [25] (electronic supplementary material, video S2). Unfortunately for the male in this case, the female was not receptive to the courtship and the interaction ended in the female cannibalizing the male (figure 6). In the last instance, when the female and male were comparable in size, we observed less interaction. Both individuals slowly extended and retracted each raptorial foreleg towards the other (known as ‘sparring’; [25]), but after 50 min both individuals reduced activity altogether and did not interact for the remainder of the night (electronic supplementary material, video S3). These anecdotal observations suggest that aggression and cannibalism are real risks, and that they might be particularly severe for individuals that are substantially smaller than their mate.

The overall extreme variation in both sexes revealed in our study suggests that any strong selection pressure on females or males to be a certain size may be overshadowed by other selection pressures that are unique to mantidfly natural history. As described above, we expect there to be strong fitness benefits to large body size in terms of reducing risks of aggression and sexual cannibalism during mating. Given that our data here suggest that the egg sac that a mantidfly enters as a larva may greatly influence its adult body size, we might predict that mantidfly larvae should make careful decisions about the spiders they board or the egg sacs they enter. While this may be the case (and studies are currently underway to examine this), there are also factors that might limit their choosiness. For example, there might be intense competition among siblings that hatch simultaneously from batches of hundreds or thousands of eggs; this may simply favour dispersal over careful assessment of nearby spiders or eggs. Moreover, there are likely to be numerous (not yet studied) challenges associated with successfully locating spider eggs at precisely the right developmental stage to consume, while mitigating predation risks, all within the short time span of the 1st instar stage. Similar to what we suspect for mantidflies, recent studies in other animals have explored how factors other than sexual selection pressure can affect patterns and plasticity in body size [4,61–64]. For mantidflies, and other insects more generally, variation in body size might affect the energetics of flight to find mates, food or appropriate habitats (see [65]). Despite the various forces shaping body size, we suspect that mantidflies are unique in that relative body size among pairs of individuals is extremely important for avoiding aggression or cannibalism during mating in a way that differs from most other animals. The intriguing and unique life history of mantidflies makes this group of insects a powerful asset in understanding how and why animals are certain sizes and the interplay between body size, sexual size dimorphism and behaviour.

## Data Availability

All data have been deposited in Dryad and are available here: https://doi.org/10.5061/dryad.mkkwh712n [66]. Electronic supplementary material is available online at [67].
